# A184 HCPE CONTRIBUTES TO THE HOST INFLAMMATORY RESPONSE TO *HELICOBACTER PYLORI*

**DOI:** 10.1093/jcag/gwad061.184

**Published:** 2024-02-14

**Authors:** C Creuzenet, N Salloum, J Lester, J M Takougoum, D Carroll, K Patel, C Atanassov, C Bodet, C Burucoa

**Affiliations:** Microbiology and Immunology, Western University Schulich School of Medicine & Dentistry, London, ON, Canada; Microbiology and Immunology, Western University Schulich School of Medicine & Dentistry, London, ON, Canada; Microbiology and Immunology, Western University Schulich School of Medicine & Dentistry, London, ON, Canada; Microbiology and Immunology, Western University Schulich School of Medicine & Dentistry, London, ON, Canada; Microbiology and Immunology, Western University Schulich School of Medicine & Dentistry, London, ON, Canada; Microbiology and Immunology, Western University Schulich School of Medicine & Dentistry, London, ON, Canada; Universite de Poitiers, Poitiers, Nouvelle-Aquitaine, France; Universite de Poitiers, Poitiers, Nouvelle-Aquitaine, France; Universite de Poitiers, Poitiers, Nouvelle-Aquitaine, France

## Abstract

**Background:**

*Helicobacter pylori* (HP) colonizes chronically 50% of the world population, leading to gastric ulcers or cancers in 2-10 % of infections. Due to rising antibiotic resistance, curtailing HP-induced diseases requires the identification of new anti-HP molecules that can reduce HP viability or its ability to elicit inflammation. Amongst HP’s virulence factors are the Helicobacter Cysteine-rich Proteins (Hcp) that are secreted outside the bacteria and contain Sel-Like Repeats (SLR) which support protein / protein interactions involved in signal transduction across kingdoms. Several SLR-containing bacterial proteins including HcpA are involved in bacteria / host interactions. Thus, Hcps may affect HP’s virulence via interaction of their SLRs with host proteins.

**Aims:**

We aim to elucidate the role of Hcps in HP’s virulence. Hcps were considered as immune decoys since anti-Hcp antibodies made in infected patients are unable to clear the infection. However, recent data suggest that Hcps interact with host components and may modulate the function of immune cells. However, their effects on gastric cells remain unknown and functional differences between Hcps remain to be explored.

**Methods:**

This work uses knockout mutagenesis, interactions with gastric cells, transcriptomics, and microscopy.

**Results:**

We focus on HcpE, the largest Hcp that may interact with many host proteins via its 9 SLRs motifs. We showed that HcpE is secreted outside HP in 2 lab strains and that ~ 30% of secreted HcpE was comprised in outer membrane vesicles that may deliver HcpE into eukaryotic cells by membrane fusion. We also showed that the DiSulfide Bond forming protein DsbK is essential for HcpE production and secretion. Here, we demonstrate the production and secretion of HcpE by clinical isolates *in vitro*, and its production *in situ* in infected patients. Using our HcpE and DsbK knockout mutants, we investigate the role of HcpE and other Hcps in interactions with human gastric cells *in vitro* and in *ex vivo* gastric explants, showing a role in inflammation activation. Finally, we demonstrate their role in murine gastric colonization and in murine splenocytes activation. Overall, our data show a role of Hcps in host / pathogen interactions largely due to immuno-modulatory functions and a role in gastric colonization, with a predominant role of HcpE over other Hcps.

**Conclusions:**

The findings open up possibilities to use Hcps or the gate keeper DsbK that controls their production as novel therapeutic targets to alleviate the burden of HP-associated disease.This could benefit the ~6 million patients who develop gastric ulcers or cancers each year worldwide due to HP infection, also saving billions of annual health care costs.

**Funding Agencies:**

Western University

